# Evaluation of the clinical characteristics and outcomes of patients admitted to intensive care units after the Kahramanmaras (Türkiye) earthquake: a multicenter observational study

**DOI:** 10.3389/fmed.2025.1517344

**Published:** 2025-02-28

**Authors:** Burcin Halacli, Goksel Guven, Esat Kivanc Kaya, Mehmet Yildirim, Nihal Deniz Bulut Yuksel, Gamze Kocak, Kadir Bulut, Derful Gulen, Begum Erdemir Sullu, Banu Kilicaslan, Asir Eren Seven, Emin Gemcioglu, Meltem Simsek, Recep Civan Yuksel, Sahin Temel, Ahmet Safa Kaynar, Kamil Deveci, Nilgun Alptekinoglu Mendil, Emre Aydin, Birkan Ulger, Aliye Esmaoglu, Nazlihan Boyaci Dundar, Ebru Ortac Ersoy, Kursat Gundogan, Seda Banu Akinci, Arzu Topeli

**Affiliations:** ^1^Division of Intensive Care Medicine, Department of Internal Medicine, Hacettepe University Faculty of Medicine, Ankara, Türkiye; ^2^Internal Medicine Intensive Care Unit, Ministry of Health, Mersin City Hospital, Mersin, Türkiye; ^3^Intensive Care Unit, Ministry of Health, Basaksehir Cam Sakura City Hospital, Istanbul, Türkiye; ^4^Division of Intensive Care Medicine, Department of Anesthesiology and Reanimation, Hacettepe University Faculty of Medicine, Ankara, Türkiye; ^5^Department of Internal Medicine, Ministry of Health, Etlik City Hospital, Ankara, Türkiye; ^6^Division of Intensive Care Medicine, Department of Internal Medicine, Erciyes University Faculty of Medicine, Kayseri, Türkiye; ^7^Intensive Care Unit, Ministry of Health, Kayseri City Hospital, Kayseri, Türkiye; ^8^Intensive Care Unit, Ministry of Health, Mersin City Hospital, Mersin, Türkiye; ^9^Division of Intensive Care Medicine, Department of Internal Medicine, Dicle University Faculty of Medicine, Diyarbakir, Türkiye; ^10^Anesthesiology and Reanimation Intensive Care Unit, Ministry of Health, Kayseri City Hospital, Kayseri, Türkiye; ^11^Division of Intensive Care Medicine, Department of Anesthesiology and Reanimation, Erciyes University Faculty of Medicine, Kayseri, Türkiye; ^12^Division of Intensive Care Medicine, Department of Internal Medicine, Gazi University Faculty of Medicine, Ankara, Türkiye

**Keywords:** disaster, crush syndrome, survival, fluid balance, renal replacement therapy

## Abstract

**Introduction:**

The outcomes of patients admitted to intensive care units (ICUs) after earthquakes that occurred on the 6th of February 2023 in Türkiye are unknown. Our objective was to delineate the demographic and clinical characteristics, therapeutic approaches, and ICU outcomes of earthquake victims who were hospitalized in Turkish ICUs.

**Methods:**

This was a retrospective multicenter study of adult patients admitted to 12 ICUs across eight tertiary hospitals located in five different cities within 2 weeks after consecutive earthquakes. Clinical and laboratory data were documented at four specific time intervals: upon hospital admission and during the first, second, and third days of ICU admission. To identify independent predictors of ICU mortality, a binary logistic regression model was used for variables identified from the univariate analysis.

**Results:**

A total of 201 patients were admitted to ICUs. The median age of the entire cohort was 36 [26–54] years. 87 patients were male (43.3%), and 114 were female (56.7%). The majority of patients (79.1%) were initially admitted to the emergency department. The median duration of being trapped under the rubble was 12 [5–31] hours. The primary reason (63.7%) for ICU admission was crush syndrome. Acute kidney injury (AKI) was identified in 61.5% of patients. Of 201 patients, 184 had information regarding ICU survival. The ICU mortality rate was 10%. A five-year increase in age, the presence of crush syndrome, and the requirement for vasopressor therapy during ICU care were independently associated with increased ICU mortality rates, while an increase of one point in the Glasgow Coma Scale (GCS) score was favorable for ICU mortality.

**Conclusion:**

This study demonstrated that crush syndrome accounted for 63.7% of the reasons for ICU admissions. The ICU mortality rate was recorded as 10%. Noteworthy independent risk factors for mortality were the presence of crush syndrome, increased age, vasopressor treatment and lower GCS score.

## Introduction

On February 6^th^, 2023, the southeastern region of Türkiye, with its epicenter located in Kahramanmaras, experienced two consecutive devastating earthquakes with magnitudes of 7.7 and 7.6 on the Richter scale that left a trail of destruction and human suffering in their wake. Officially, 115,353 people were injured, and 50,783 died, according to the report of The Turkish Ministry of Interior Disaster and Emergency Management Presidency. Moreover, 37,984 buildings collapsed, which stands out as Türkiye’s most devastating earthquake in the last century (1).

Early after an earthquake, deaths primarily occur due to direct trauma to major organs (2). However, rescued victims can be at significant risk for morbidity and mortality due to direct or indirect consequences of crush injuries. These patients need specialized care and resources for immediate and aggressive treatment, such as fluid therapy, surgical intervention, and hemodialysis, to prevent crush syndrome and life-threatening complications (3–6).

Crush syndrome, which is the leading cause of mortality among rescued victims, is a traumatic rhabdomyolysis caused mainly by direct or indirect trauma to muscle-rich body parts, such as limbs or torso, resulting in the compression and destruction of striated muscle cells (7, 8). Once the blood supply of these tissues is restored, the cell products, notably myoglobin, potassium, urate, and phosphate, are released into the systemic circulation, which causes acute kidney injury (AKI), electrolyte and metabolic disturbances, and multiple organ dysfunction syndrome (9).

While immediate rescue and relief efforts were critical in the aftermath of the earthquake, the healthcare needs of survivors, especially those requiring intensive care, have been largely overlooked. In this study, we aimed to describe the demographic and clinical profiles, treatment modalities, and intensive care unit (ICU) outcomes of earthquake victims admitted to ICUs in Türkiye.

## Materials and methods

### Study design and data

**Figure 1:**
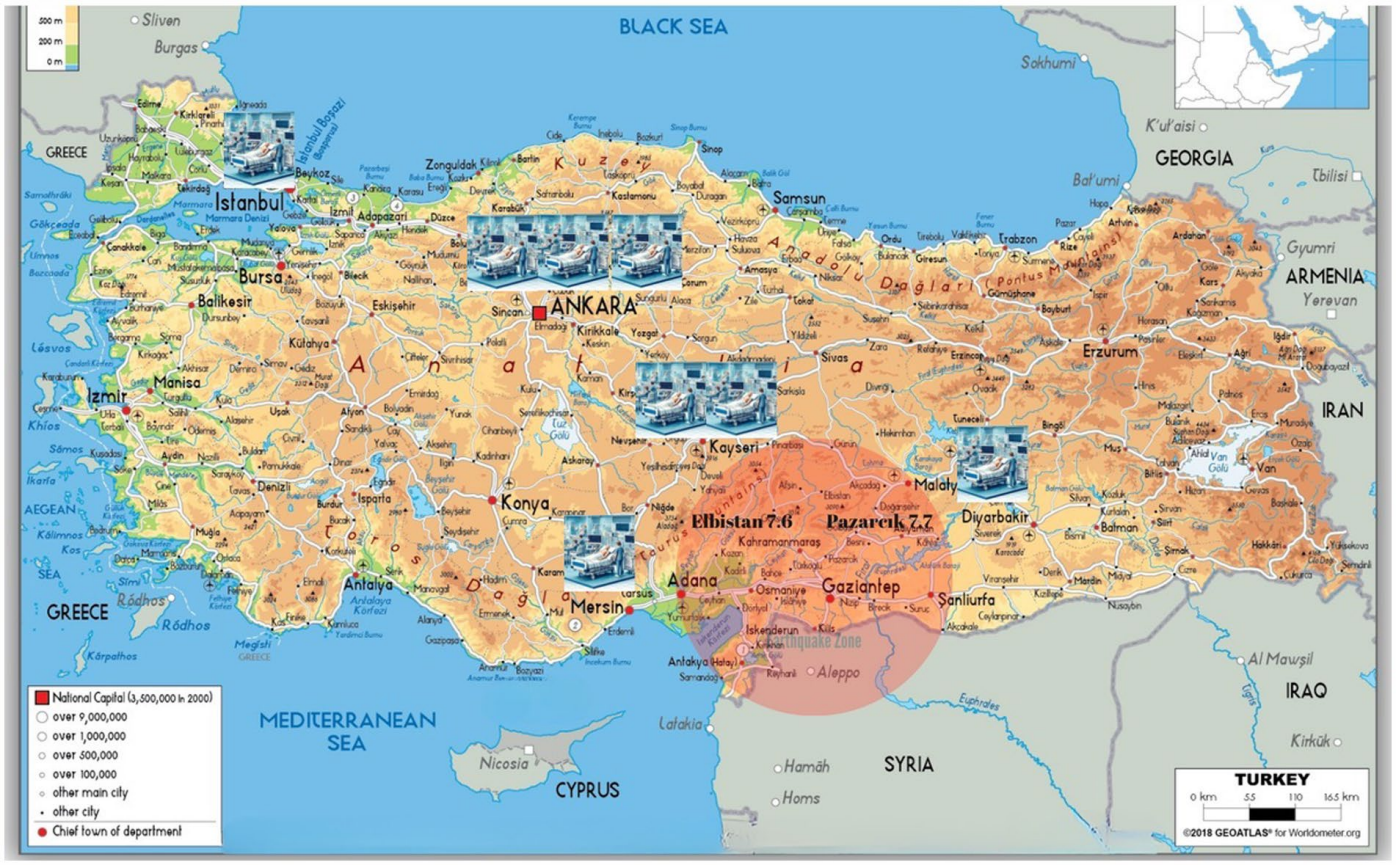
Türkiye map illustrating earthquake zone and the location of referreal hospitals. Patient data from 12 intensive care units across eight tertiary care hospitals were gathered retrospectively for those admitted within 2 weeks following the earthquakes. The hospitals included three from Ankara and one from Istanbul, located farther away from the earthquake epicenter, and two from Kayseri, along with one each from Diyarbakir and Mersin, which are relatively close to the earthquake zone. Türkiye map was gathered from www.worldometers.info webpage. The illustations and photo creations were carried out by www.canva.com and ChatGPT4.0.

The data of all consecutive patients admitted to the 12 ICUs of eight tertiary care hospitals within 2 weeks of the earthquake were collected retrospectively. These hospitals were 3 from Ankara and 1 from Istanbul, which are far from the epicenter of the earthquake, and 2 from Kayseri, 1 from Diyarbakir and Mersin, which are quite close to the earthquake zone (Figure 1). All critically ill adults (≥18 years) admitted to those ICUs due to direct or indirect effects of earthquake-associated trauma were included in this study.

A standardized data collection process was implemented using an Excel form. Demographic data, comorbidities, injury types, and patient outcomes were all entered by the responsible investigators for each hospital. After data entry, all information was anonymized and compiled for analysis. Variables were collected from the hospitals’ electronic health record systems and patient charts. Demographic data were recorded during hospital admission. The clinical and laboratory data were recorded at four time points: at hospital admission and on the first, second, and third days of the ICU stay. The hospital admission variables were the first measured variables in those referral centers. Vital signs, urine output, and laboratory test results were recorded for each time point. However, fluid therapy before hospital admission, the location of the trauma on the body, respiratory and hemodynamic support, Glasgow Coma Scale (GCS) score, and Revised Trauma Score (RTS) were recorded at hospital admission. Acute Physiology and Chronic Health Evaluation (APACHE) II, Sequential Organ Failure Assessment (SOFA) and modified Nutrition Risk in Critically Ill (mNUTRIC) scores were also recorded at ICU admission. Due to differences in the reference values at each hospital, creatinine kinase (CK) and troponin levels were considered to indicate CK levels greater than 10 times the local reference range and any abnormalities above the local reference range, respectively. All patients were followed up until they were discharged from the hospital, transferred to another hospital, or died.

### Definitions

Crush injury was diagnosed as swollen limbs and a history of limb compression. Crush syndrome was diagnosed if the crush injury was accompanied by acute kidney injury (AKI) and/or multiple organ failure (10, 11). The Kidney Disease Improving Global Outcome (KDIGO) criteria were used for AKI diagnosis (12). The presence of AKI was determined during the pre-ICU and ICU admission periods. However, patients without previously known chronic kidney disease (CKD) who presented with a creatinine value of at least 0.3 mg/dL above the upper limit of the normal range or who required hemodialysis were considered to have AKI. If the patient previously had CKD, the admission creatinine level was compared to the most recent creatinine level measured before the earthquake using national electronic health records. The attending nephrologists and consultant intensivist decided on the indication for intermittent hemodialysis (IHD) and the initiation of continuous renal replacement therapy (CRRT).

### Statistical analysis

Descriptive data are presented as the mean (standard deviation) and median (interquartile range-IQR) for continuous variables according to the normality of the distributions, which was assessed by the Kolmogorov–Smirnov test. Categorical variables are shown as frequencies. To evaluate the trend of continuous variables between admission and the first 3 days of ICU stay, analysis of variance (ANOVA) or the Friedman test was used, as appropriate. Categorical variables were compared using the chi-square test. Significant variables without interconnected variables identified from the univariate analysis were included in the binary logistic regression model to identify independent predictors of ICU mortality, and the results are presented as odds ratios (ORs) and 95% confidence intervals (CIs). The adequacy of the model fit was evaluated using the Hosmer–Lemeshow goodness-of-fit test. All tests were two-tailed, and *p* < 0.05 was considered to indicate statistical significance. All the statistical analyses were performed using the IBM Statistical Package for Social Sciences (SPSS) program version 29.0 (IBM Corp., Armonk, NY, USA) software.

## Results

Two hundred and one (*N* = 201) earthquake victims were admitted to twelve ICUs across eight referral hospitals within 2 weeks following the earthquake. Table 1 shows demographic data and baseline characteristics. The median age of the whole cohort was 36 [26–54] years. There were 87 males (43.3%) and 114 females (56.7%). The emergency department was the first admission unit for the majority (79.1%) of the patients. The duration of being stuck under the rubble was 12 [5–31] hours, and the length of stay in the first hospital was 28.5 [15–72] hours. The major reason for ICU admission was trauma-related injuries, mainly crush syndrome (63.7%). Information regarding prehospital fluid therapy was obtained for 103 patients, which was 2.0 [1.0–2.5] liters (L) before transfer to the referral hospital. AKI was detected in 61.5% of the 187 patients who had AKI data available before ICU admission. Furthermore, data regarding the necessity for hemodialysis were obtained for 194 patients, 41 (21.1%) of whom required intermittent hemodialysis (IHD) during their stay at the initial hospital. Table 2 shows the temporal changes in laboratory findings from the onset of hospital admission over the first 3 days for 193 patients. Eight patients had data only during ICU admission and not during the initial 3 days in the ICU.

**Table 1:** Characteristics of patients before ICU admission.

Characteristics	(*n* = 201)
Age, year (IQR)	36 [26–54]
Gender, *n* (%)	
Male	87 (43.3)
Female	114 (56.7)
Comorbidity, *n* (%)	57 (28.3)
Hypertension	27 (13.4)
Diabetes Mellitus	20 (9.7)
Cardiovascular disease	12 (5.9)
Hypothyroidism	10 (4.9)
Respiratory disease	7 (3.4)
Cerebrovascular disease	5 (2.4)
Chronic kidney disease	4 (2.0)
Unit from where the patient was transferred to the ICU, *n* (%)	
Emergency department	159 (79.1)
Direct transfer from the field	27 (13.4)
Ward	10 (5.0)
Operation room	5 (2.5)
Time stuck under the rubble, hours (IQR)	12.0 [5–31.5]
Length of stay at the prior health center, hours (IQR)	28.5 [15–72]
Fractures and crush injuries, *n* (%)	
Crush injury of extremities	155 (77.1)
Thorax injury	73 (36.3)
Limb fracture	72 (35.8)
Spine fractures	53 (26.3)
Maxillofacial injury	38 (18.9)
Neck injury	29 (14.4)
Abdominal injury	25 (12.4)
Skull bone fractures	11 (5.4)
Brain injury	7 (3.5)
Crush syndrome, *n* (%)	128 (63.7)
Prehospital fluid therapy (*n* = 103), milliliters, (IQR)	2000 [1000–2,500]
AKI (*n* = 187), *n* (%)	115 (61.5)
Hemodialysis (*n* = 194), *n* (%)	41 (21.1)
Fasciotomy, *n* (%)	60 (29.9)
Surgery, *n* (%)	89 (44.2)

IQR, interquartile range.

**Table 2:** Temporal trend of laboratory parameters upon admission and during the initial 3 days in the ICU.

Parameters	Hospital admission	ICU Day 1	ICU Day 2	ICU Day 3	*p* value
Kidney function tests					
Creatinine, mg/dL (*n* = 151)*	1.96 [0.72–3.9]	1.81 [0.61–3.8]	1.59 [0.72–3.63]	1.31 [0.5–3.6]	**<0.001**
BUN, mg/dL, (*n* = 135)*	37 [22–61]	34 [15–54]	28 [13–46]	24 [12–43]	**<0.001**
Electrolytes					
Na, mEq/L (*n* = 151)*	135 [131–139]	136 [132–140]	137 [133–140]	137 [133–140]	**<0.001**
K, mEq/L (*n* = 151)*	4.7 [3.9–5.9]	4.4 [3.8–5.0]	4.0 [3.6–4.6]	3.96 [3.6–4.3]	**<0.001**
P, mg/dL (*n* = 106)*	5.1 [3.0–7.0]	4.1 [2.6–6.2]	3.5 [2.5–5.5]	3.5 [2.4–5.4]	**<0.001**
Ca, mg/dL (*n* = 148)*	7.6 [6.8–8.3]	7.1 [6.5–7.7]	7.1 [6.6–7.6]	7.2 [6.7–7.7]	**<0.001**
Liver function tests					
AST, U/L (*n* = 147)*	485 [130–955]	318 [93–796]	259 [80–541]	190 [65–398]	**<0.001**
ALT, U/L (*n* = 146)*	157 [56–369]	127 [45–278]	92 [38–223]	84 [35–162]	**<0.001**
Total bilirubin, mg/dL (*n* = 145)*	0.6 [0.4–0.94]	0.5 [0.32–0.72]	0.48 [0.3–0.68]	0.5 [0.32–0.7]	**<0.001**
Albumin, g/dL (*n* = 122)*	2.8 [2.3–3.5]	2.5 [2.1–2.9]	2.3 [2.0–2.6]	2.3 [2.0–2.6]	**<0.001**
Cardiac and muscle markers					
CK-MB, ng/mL (*n* = 62)*	21.5 [7.2–229.1]	84.4 [28.9–779.5]	32.3 [6.9–215.8]	24.5 [7.5–126.5]	**<0.001**
High Troponin^δ^, ng/mL (*n* = 79) (*n*, %)	39 (49)	50 (63)	39 (49)	31 (39)	**<0.001**
Myoglobin, μg/L (*n* = 52)	1000 [827–4105]	1058 [1000–4105]	1000 [328–4105]	627 [181–1000]	**<0.001**
CK > 10 times, U/L (*n* = 126) (*n*, %)	111 (88)	108 (86)	100 (79)	94 (75)	**<0.001**
LDH, U/L (*n* = 115)	847 [526–1992]	888 [468–159]	680 [439–1200]	612 [436–983]	**<0.001**
Complete blood count					
Hb, g/dL (*n* = 151)	11.8 ± 3.4	9.9 ± 2.6	9.1 ± 2.6	8.7 ± 1.5	**<0.001**
Htc, % (*n* = 151)	35.5 ± 10.3	29.8 ± 7.6	26.5 ± 5.7	25.8 ± 5.0	**< 0.001**
Leukocyte, 10^3^/μL (*n* = 151)*	14.7 [11.9–22.3]	12.7 [9.9–16.6]	12.0 [9.4–15.5]	11.3 [8.8–15.6]	**< 0.001**
Platelet, 10^3^/μL (*n* = 151)*	210 [168–291]	181 [138–254]	155 [117–222]	162 [111–250]	**<0.001**
Coagulation tests					
INR (*n* = 126)*	1.15 [1.0–1.36]	1.11 [1.0–1.3]	1.11 [1.0–1.28]	1.1 [1.0–1.28]	0.20
aPTT, second (*n* = 125)*	27.8 [24.6–34.5]	28.7 [25.1–35.6]	29.6 [25.3–35.0]	29.2 [24.9–33.6]	**0.03**
D-dimer, mg/L (*n* = 58)*	4.8 [2.7–11.1]	4.8 [2.5–9.5]	5.1 [2.9–9.3]	5.5 [3.4–9.5]	0.07
Fibrinogen, mg/dL (*n* = 80)	472 ± 196	490 ± 186	490 ± 200	480 ± 212	0.65
Arterial blood gas tests					
pH (*n* = 139)*	7.36 [7.28–7.42]	7.4 [7.33–7.45]	7.4 [7.36–7.44]	7.42 [7.38–7.46]	<**0.001**
HCO_3_, mmol/L (*n* = 139)*	19.2 [15.6–23.3]	21.6 [18.8–25.0]	23.4 [21.0–26.0]	24.2 [21.8–27.0]	**< 0.001**
pO_2_, mmHg (*n* = 75)*	91 [78–119]	87 [73–116]	95 [76–122]	95 [78–120]	0.78
pCO_2_, mmHg (*n* = 75)*	33 [28–38]	36 [31–39]	37 [34–41]	37 [33–40]	**0.004**
SaO_2_, % (*n* = 74)*	96.5 [94–98]	97 [94–98.6]	97 [95–98]	97 [95–99]	0.86
Lactate, mmol/L (*n* = 139)*	1.8 [1.2–2.8]	1.4 [0.9–2.2]	1.3 [0.9–1.8]	1.26 [0.9–1.7]	**<0.001**
Anion Gap, mEq/L (*n* = 132)*	11.5 [8.2–16.6]	6.7 [8.2–12.9]	6.8 [8.2–11.7]	6.0 [8.2–11.2]	**<0.001**
SID, mEq/L (*n* = 136)*	34.6 [29.4–37.9]	34.0 [31.6–37.3]	35.3 [32.0–37.7]	35.3 [33.0–37.9]	0.14
BE, mEq/L (*n* = 136)*	−5.6 [−10.0–−0.4]	−3.3 [−6.6–1.3]	−0.9 [−4.3–−2.8]	0.8 [−2.7–3.4]	**<0.001**

^*^Median [IQR], others mean ± SD; BUN: blood urea nitrogen, AST, aspartate aminotransferase; ALT, alanine transaminase; Na, sodium; K, potassium; P, phosphorus; Ca, calcium; iCa, ionized calcium; CK-MB, creatine kinase-myoglobin binding; LDH, lactate dehydrogenase; Hb, hemoglobin; Hct, hematocrit; INR, international normalized ratio; aPTT, activated partial thromboplastin time; HCO3, bicarbonate; pO_2_, partial pressure of oxygen; pCO_2_, partial pressure of carbon dioxide; SaO_2_, oxygen saturation; SID, strong ion difference; BE, base excess; CK, creatine kinase.

^δAbove the local reference range.^

Eight patients had only ICU admission data without any follow-up data within 3 days of ICU admission.Bold values mean statistically significance.

### Fluid balance and hemodynamic evaluation

The amount of fluid intake and output (urine and nonurine output) were as follows for the first 3 days of the ICU stay: 4800 [2858–7140] ml and 1500 [500–3000] ml for the first day, 4615 [3400–6050] ml and 2400 [1200–3500] ml for the second day, and 4000 [3150–5400] ml and 2500 [1280–4200] ml for the third day. There was heterogeneity in the type of fluid infused in the ICU. During the first ICU day, normal saline (96.3%) was the most preferred fluid, followed by dextrose solutions (71%), balanced crystalloid fluids (32.5%), and albumin (19.6%). Among all patients, 37% received HCO_3_ infusion for urine alkalization, 36.3% required renal replacement therapy (RRT), 21.4% were administered furosemide, and 6% received mannitol infusion. In total, 112 patients received blood products. Among these patients, 110 patients received red blood cells (2 [0–4]), 28 patients received platelets, 48 patients received fresh frozen plasma, and 6 patients received cryoprecipitate transfusions.

Of the 101 patients, fluid responsiveness was assessed solely based on clinical examination, whereas in the remaining 100 patients, at least one of the following methods was utilized: cardiac point-of-care ultrasonography (65 patients), central venous pressure (CVP) monitoring (63 patients), capillary refilling time assessment (22 patients), passive leg raising test (11 patients), and/or thermodilution technique (2 patients).

### Comparison of variables between survivors and nonsurvivors

Of the 201 patients, 184 had information regarding ICU survival since 17 were transferred to other hospitals for any reason and were lost to follow-up. Five patients were transferred on the second day, and three were transferred on the third day of the ICU stay. The ICU mortality rate was 10%. Table 3 shows the clinical and ICU variables related to ICU mortality.

**Table 3:** Comparison of patient demographics, clinical characteristics, and outcomes between ICU survivors and nonsurvivors.

	All patients (184)	Survivors (*n* = 164)	Nonsurvivors (*n* = 20)	*p* value
Age, year*(minimum-maximum)	36 [26–56] [18–85]	36 [26–56] [18–85]	45 [34–59] [20–83]	0.18
Female sex, *n* (%)	101 (54.9)	73 (44.5)	10 (50)	0.64
Time stuck under the rubble, hours*	12 [5–30]	12 [4–30]	15 [9–24]	0.21
Length of stay at the prior health center, hours*	29 [15–72]	29 [15–68]	34 [10–167]	0.81
Clinical findings during admission				
APACHE II score^†^	17 ± 7.8	16 ± 7.1	27 ± 8.1	**<0.001**
SOFA score*	4 [1–6]	3 [1–5]	9 [5–12]	**<0.001**
GCS*	15 [14–15]	15 [15–15]	8 [3–14]	**<0.001**
RTS*	12 [12–12]	12 [12–12]	8 [6–12]	**<0.001**
Modified NUTRIC score*	2 [1–3]	2 [1–3]	5 [3–6]	**<0.001**
MAP<65 mmHg, *n* (%)	13/165 (7.9)	5/146 (3.4)	8/19 (42.1)	**<0.001**
Heart rate > 100/min, *n* (%)	97/174 (55.7)	85/155 (54.8)	12/19 (63.2)	0.49
Respiratory rate > 20/min, *n* (%)	103/164 (62.8)	92/147 (62.6)	11/17 (64.7)	0.86
SO_2_ < 90%, *n* (%)	12/172 (7.0)	9/153 (5.9)	3/19 (15.8)	0.11
AKI, *n* (%)	103/170 (60.6)	86/164 (52.4)	17/20 (85.0)	**0.02**
Crush syndrome, *n* (%)	116/184 (63)	99/164 (59.6)	17/20 (85.0)	**0.03**
Intermittent hemodialysis, *n* (%)	38/179 (21.2)	33/164 (20.1)	5/20 (25.0)	0.66
Total I.V fluid administered, milliliter*	2000 [1250–2675]	2000 [1200–2500]	2440 [2000–3500]	**0.055**
Compartment syndrome, *n* (%)	116/184 (63.0)	59/164 (36.0)	9/20 (45.0)	0.43
Fasciotomy, *n* (%)	54/183 (29.5)	46/164 (27.7)	8/20 (40.0)	0.52
Parameters during ICU stay				
High flow nasal oxygen therapy, *n* (%)	20/184 (10.9)	19/164 (11.6)	1/20 (5.0)	0.63
NIMV, *n* (%)	18/184 (9.8)	16/164 (9.8)	2/20 (10.0)	0.97
IMV, *n* (%)	63/184 (34.2)	46/164 (28.0)	17/20 (85.0)	**<0.001**
Vasopressor support, *n* (%)	39/184 (21.2)	21/164 (12.8)	18/20 (90.0)	**<0.001**
Blood transfusion, *n* (%)	105/184 (57.1)	94/164 (57.3)	11/20 (55.0)	0.84
Nosocomial infection, *n* (%)	45/182 (24.7)	41/164 (25.0)	4/20 (20.0)	0.77

*median [IQR], ^†^mean ± SD, others *n* (%); APACHE, Acute Physiology and Chronic Health Evaluation; SOFA, Sequential Organ Failure Assessment; GCS, Glasgow Coma Scale; RTS, Revised Trauma Score; NUTRIC, Nutrition Risk in Critically Ill; MAP, mean arterial pressure; AKI, acute kidney injury; I.V, intravenous; NIMV, noninvasive mechanical ventilation; IMV, invasive mechanical ventilation.

The survival of 17 patients was not detected due to an unknown follow-up due to transfer to different hospitals.Bold values mean statistically significance.

The ICU admission scores were significantly worse in nonsurvivors. The presence of tachycardia, tachypnea, and hypoxemia at hospital admission did not significantly differ between survivors and nonsurvivors; however, arterial hypotension was more frequent in nonsurvivors (3.4% vs. 42.1%, *p* < 0.001). The need for vasoactive agents due to hypotension was greater in the nonsurvivors during their ICU follow-up (12.8% vs. 90%, *p* < 0.001).

AKI (52.4% vs. 85%, *p* = 0.019) and crush syndrome (59.6% vs. 85.0%, *p* = 0.031) at hospital admission were more frequent in the nonsurvivors. On the other hand, there was no significant difference between the two groups regarding the need for hemodialysis before admission to the referral hospital (20.1% vs. 25%, *p* = 0.663). During their follow-up in the ICU, 89 patients underwent hemodialysis. Among these patients, 61 received IHD, 11 received continuous renal replacement therapy (CRRT), 16 received a combination of IHD and CRRT, and one patient underwent peritoneal dialysis (PD). The median number of IHD sessions and duration of CRRT were 3 (IQR, [2–6]) sessions and 72 (IQR, [48–120]) hours, respectively.

Among the patients, 63 received invasive mechanical ventilation (IMV) support, 18 received noninvasive mechanical ventilation (NIMV) support, and 19 received high-flow nasal oxygen (HFNO) therapy. The nonsurvivors needed more IMV support than did the survivors (28% vs. 85%, *p* < 0.001). However, the use of NIMV and HFNO did not significantly differ between the groups.

### Associations between hospital and ICU admission variables and mortality

After eliminating interconnected variables (AKI during admission, APACHE II score, admission RTS, SOFA score and modified NUTRIC score having same substances in their structures), univariate logistic regression analysis was performed to determine the determinants of ICU mortality. Multivariate logistic regression analysis was performed for age (with a 5-year increase), GCS score (with a 1-point increase), presence of crush syndrome upon hospital admission, requirement for IMV, and administration of vasopressor therapy during ICU care. The results revealed that the occurrence of crush syndrome, a 5-year increase in age, and the need for vasopressor therapy during ICU treatment were independently correlated with increased ICU mortality. A 1-point increase in the GCS score is favorable for ICU mortality (Table 4).

**Table 4:** Multivariate logistic regression model for ICU mortality.

Variables	OR (95% CI)	*p* value
Age (per 5 years increase)	1.31 (1.05–1.63)	**0.015**
GCS at admission (per 1-point increase)	0.84 (0.73–0.97)	**0.016**
Crush syndrome at admission	9.24 (1.09–78.55)	**0.042**
Vasopressor support during ICU stay	34.79 (6.01–201.4)	**<0.001**
Invasive mechanical ventilation during ICU stay	0.85 (0.11–6.82)	0.87

ICU, intensive care unit; GCS, Glasgow Coma Scale.Bold values mean statistically significance.

## Discussion

This study demonstrated that crush syndrome is the leading cause of ICU admission, and its incidence is greater in the 2023 Kahramanmaras earthquake than in previous reports. The mortality rate in the ICU was 10%. Crush syndrome, older age, lower GCS score at admission and the need for vasopressors are independent risk factors for ICU mortality after an earthquake.

The 2023 Kahramanmaras earthquake, a sudden and devastating event, raised significant challenges to the healthcare system and prompted the need for a comprehensive study on the intensive care follow-up of patients affected by this natural disaster. To our knowledge, this study is the first multicenter study assessing earthquake victims from the intensive care perspective from real life, including a relatively large number of earthquake victims.

We reported the highest incidence of crush syndrome in the literature (63.7%). In parallel with our study, the incidence of crush syndrome was reported to be 50% in patients transferred to the ICU after the 1995 Hanshin-Awaji earthquake in Japan (13). Crush syndrome was seen 0.9% after the 2010 Yushu earthquake, 5% after the 2008 Wenchuan earthquake, 14% after the Tangshan earthquake, and 30% after the 1999 Marmara earthquake (14–16). The main underlying reason regarding this concrete diffrenece is that our study is restricted to patients admitted to the ICU rather than including all hospitalized patients. Other factors contributing to such considerable variation in crush syndrome incidence could include the timing of the earthquake, the construction of buildings in the affected area, the time stuck under the rubble, first aid, and rescue possibilities (7, 17, 18). Because the earthquake in Türkiye hit in the early morning during sleep, people did not have time to escape outside and were stuck under the concrete and stone buildings. Unfortunately, the patients were also exposed to cold and hypothermia.

The ICU mortality rate was 10% in this study. Data on the mortality of patients admitted exclusively to ICUs following earthquake disasters remain limited. In a subgroup analysis of a study conducted by Tanaka et al. on survivors of the 1995 Hanshin-Awaji earthquake requiring hospital admission, the mortality rate among those admitted to the ICU was reported as 13.4% (13). The lowest mortality in our study can be attributed to several factors. First, remarkable progress has transpired within the field of intensive care, particularly over the last decade. Primarily driven by the dynamic use of hemodynamic monitoring techniques, there has been a notable shift from the previously used liberal fluid therapy to more restrictive regimes (19–21). Especially by integrating echocardiography into intensive care practice, standard goal-directed fluid therapy protocols have evolved into personalized fluid therapy algorithms (22–24). In the present study, echocardiography was used to assess fluid responsiveness in almost one-third of our patients. The primary reason for the inability to use echocardiography in many of the remaining patients was the restriction of obtaining high-quality images for measuring cardiac output. Due to severe traumatic injuries in the lower extremities, the passive leg-raising test could be applied in a limited group of patients. Similarly, the limited use of thermodilution techniques in only two patients may be attributed to the unsuitability of femoral arterial access on a case-by-case basis. Although not used alone to assess fluid responsiveness, CVP measurement has been employed in approximately one-third of patients. In current intensive care practices, this technique is used in addition to other methods due to its low sensitivity and specificity for assessing fluid responsiveness (25). It should be noted that the reliability of CVP measurements is controversial in patients with increased intraabdominal pressure, which is an expected complication in earthquake victims (26).

Compared to nontrauma ICU patients, earthquake victims could be more susceptible to tissue edema, particularly due to preexisting damaged tissue. Therefore, nonliberal fluid regimes might help to prevent an increase in mortality in our population. In our study, the amount of fluid administered on the first day in the ICU was 4.8 [IQR, 2.8–7.1] liters. Similarly, after the 1999 Marmara earthquake, Sever et al. reported that the amount of fluid administered to earthquake patients was 5.1 ± 1.7 liters (27). Additionally, similar to our study, Sever et al. revealed that the most commonly used fluids were normal saline, dextrose solution, mannitol, and bicarbonate (27). However, it is important to emphasize that balanced crystalloid solutions were also used in one-third of the patients in this study. Despite containing low amounts of potassium, balanced crystalloid solutions have pH, chloride, and strong ion difference (SID) values at relatively physiological limits compared to those of normal saline (28). Although the data from our study do not specify which type of fluid may positively influence mortality and morbidity, and normal saline remains the traditionally accepted solution for crush injuries, our findings suggest that balanced crystalloid solutions may be safely utilized in carefully monitored patients. Excess fluid negatively affects tissue oxygenation by inducing iatrogenic hemodilution, disrupting tissue and cell architecture, impairing lymphatic drainage, increasing diffusion distance, and decreasing functional capillary density (29, 30).

Crush syndrome is the most significant cause of mortality in patients rescued from the rubble of an earthquake disaster (7, 31). In line with previous studies, the current study reported that crush syndrome was an independent risk factor for ICU mortality. Almost two-thirds of patients had crush syndrome before admission to the ICU. Of all the patients with crush syndrome, 85% underwent fasciotomy, and 93% had their first hemodialysis at their first hospital. Notably, these patients still had a high risk for mortality and needed close monitoring and ICU follow-up, even if they were clinically stable following the initial medical intervention. In parallel, after the Hanshin Awaji earthquake, Tanaka and colleagues reported increased mortality among patients with crush syndrome who were not transported from the affected hospitals to the backup hospitals (13).

Another main finding of our study is that increased age is associated with increased ICU mortality. There is a greater prevalence of comorbidities in elderly patients, which increases the likelihood of mortality. In our series, the median age of the patients was quite young (36 years [26–54]), and only 14% were ≥ 65 years old. This proportion correlates well with previous earthquake reports (14, 27, 32, 33).

Innovations in mechanical ventilation techniques, the use of CRRT in hemodynamically unstable patients, sedation, nutrition, and physiotherapy programs might have substantially contributed to the recovery of these patients. Indeed, one-third of the patients needed IMV, and half of them required NIMV and HFO support. While IHD is recommended as the primary option for hemodialysis in earthquake patients (34), 14% of patients received CRRT. The main reason for this is the presence of hemodynamic instability that results in intolerance to IHD. Nevertheless, the impact of CRRT on outcomes in these patients needs to be elucidated. The relatively low implementation of renal replacement therapy observed in our study is consistent with findings from previous earthquakes. Studies on the Bingöl-Türkiye and Haiti earthquakes have highlighted that timely and effective fluid therapy in patients with crush syndrome can significantly reduce the need for dialysis (35).

Earthquake trauma may cause a systemic response, precipitating a cascade of events resulting in multiorgan dysfunction (36). Therefore, laboratory tests of ICU patients revealed AKI, acute liver injury, elevated cardiac markers, electrolyte abnormalities, leukocytosis, acidosis, and elevated tissue perfusion parameters such as lactate. Remarkably, these indices tended to decrease during the ICU stay. Particularly after an earthquake, the development of hyperinflammation, extravasation of intracellular fluids into damaged tissues, initial restrictions to fluids, and increased insensible fluid losses lead to significant hypovolemia (34).

During an earthquake, the lower extremities are reportedly the most injured body parts. Afterward, there is a discernible predilection for an increased incidence of cranial trauma in earthquakes occurring during day hours when individuals are awake and abdomen-thorax trauma in earthquakes occurring at night (37, 38). Therefore, not surprisingly, extremity and thoracic injuries were the most frequently encountered body parts in the Kahramanmaras earthquake. In the 1999 Marmara earthquake in Türkiye, thoracic and abdominal injuries were more frequent and were associated with high mortality.

### Limitations

This study has several limitations that need to be highlighted. First, it was restricted to only 5 hospitals and 11 ICUs. Considering the large sample size and the inability to evacuate numerous critically ill patients from the earthquake-affected region, the number of patients included in our study was low, and the patients may have been relatively less critically ill, which might have caused selection bias. Another limitation is the absence of outcome data for patients transferred to different ICUs included in the study. Nevertheless, this proportion remains below 10% of the total population. In the assessment of AKI, the urine output criterion could not be utilized in patients with crush injuries in this study due to the presence of hypovolemia, insufficient intravascular volume caused by crush syndrome, and the early initiation of diuretics in some patients. Due to the retrospective study design, inherent faults and inadequacies may be possible, especially regarding the documentation of data which might caused low frequencies of utilization of emergency sonografic examinations and CRT. Moreover, the absence of an established standardized fluid protocol for crush syndrome associated with earthquakes, as well as the case-by-case determination of dialysis indications by clinicians amidst the chaotic circumstances of the disaster, necessitates caution when interpreting the outcomes for this patient group.

## Conclusion

The current study showed that the incidence of crush syndrome surpassed that of other causes for ICU admissions, with a higher incidence rate than that reported in previous studies. The mortality rate in the ICU was 10%. Factors such as crush syndrome, every 5 years increase in age, lower GCS scores upon admission and the need for vasopressors during ICU care emerged as distinct risk factors for mortality following the Kahramanmaras earthquake.

## Data Availability

The processed data is available upon reasonable request by the corresponding author.
