# Supplementary figures and images for: Evaluation of the clinical characteristics and outcomes of patients admitted to intensive care units after the Kahramanmaras (Türkiye) earthquake: a multicenter observational study
Source: Front Med (Lausanne). 2025 Feb 28;12:1517344. doi: 10.3389/fmed.2025.1517344 (PMC11907275; doi:10.3389/fmed.2025.1517344)

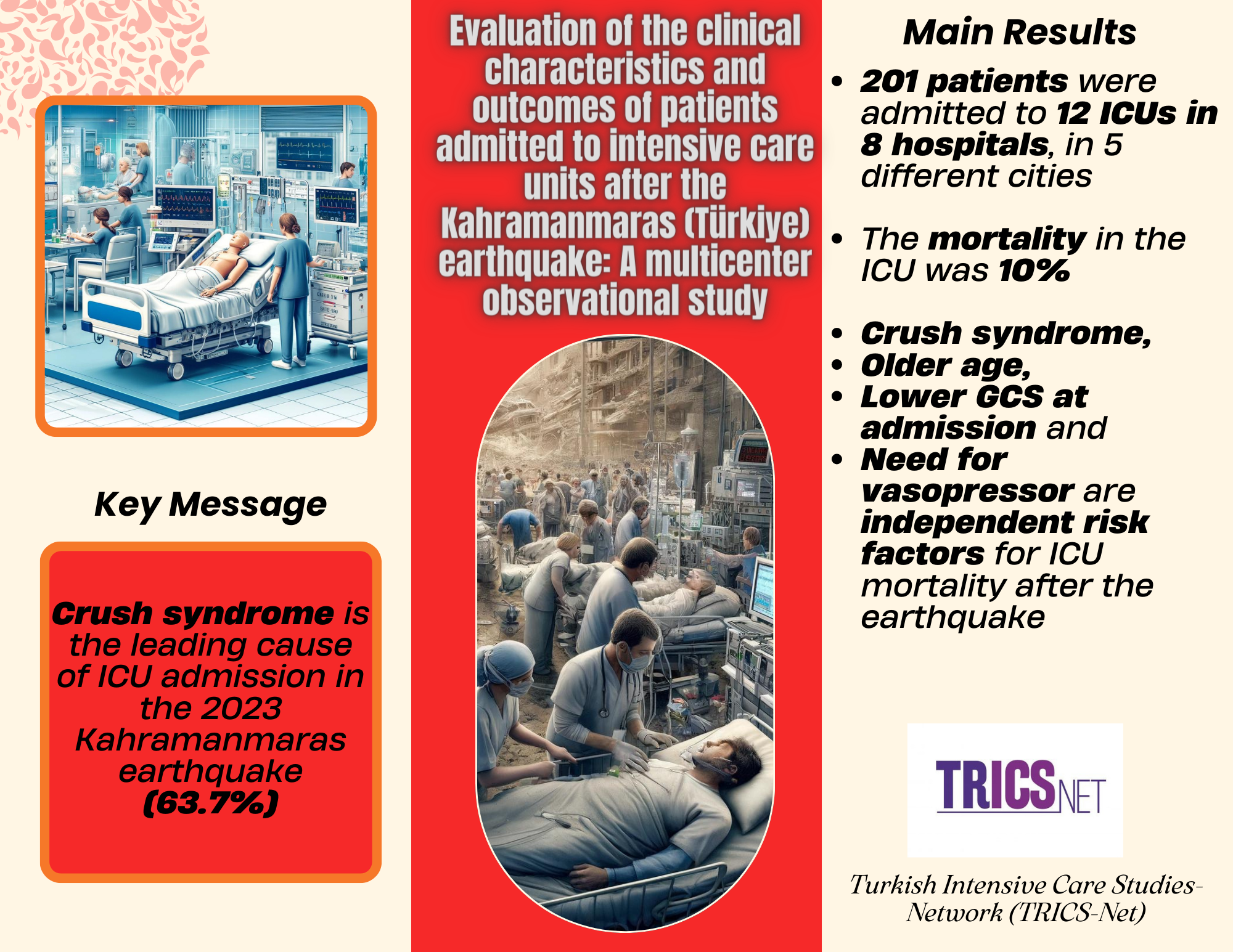

Supplement: Supplementary file 1 [file Image_1.PNG]
